# Estimating cucumber crop coefficients under different greenhouse microclimatic conditions

**DOI:** 10.1007/s00484-023-02535-y

**Published:** 2023-09-21

**Authors:** Georgios Nikolaou, Damianos Neocleous, Evangelini Kitta, Nikolaos Katsoulas

**Affiliations:** 1 Department of Agriculture Crop Production and Rural Environment, School of Agricultural Sciences, University of Thessaly, Fytokou Str., 38446 Volos, Greece; 2 Department of Natural Resources and Environment, Agricultural Research Institute, Ministry of Agriculture, 1516 Nicosia, Cyprus

**Keywords:** Evapotranspiration, Mediterranean region, Priestley-Taylor, Irrigation scheduling

## Abstract

This study aimed to determine cucumber crop coefficients under different greenhouse microclimatic conditions, parameterizing the Priestley-Taylor reference evapotranspiration model. Crop evapotranspiration was directly measured with the use of lysimeters, and crop coefficients were computed following the two-step climate FAO 56 methodology. Greenhouse compartments (i.e., cooled or uncooled) showed reference evapotranspiration differences of up to 12% in an autumn-winter crop. The results presented cucumber crop coefficient values from the initial to the late-season growth stages from 0.45 to 0.94 depending on the greenhouse climate. Based on the greenhouse hourly microclimatic variation of *K*_*C*_, it is recommended not to apply a *K*_*C*_ as a constant for transpiration estimation even at greenhouses located within the same region Regression analysis relating crop coefficients with leaf area revealed very high correlation coefficients for the equations tested. The results indicated that evapotranspiration can be modeled satisfactory based on a significant relationship between crop coefficient and simple measurements of the leaf area index (i.e., *K*_*C*_ = 0.447 × LAI).

## Introduction

Adequate prediction of crop evapotranspiration (*ET*_*C*_; i.e., the amount of water loss through soil evaporation and crop transpiration) represents a useful parameter in hydrological, climate studies, and agricultural systems (Nistor et al. 2017). More importantly, *ET*_*C*_ must be considered for irrigation scheduling allowing for potential water savings and lower environmental impacts. However, variability in climatic conditions affects *ET*_*C*_ even in the same crop species (De Medeiros et al. 2005; Mojid et al. 2015; Rahman et al. 2015; Gong et al. 2020a). Therefore, emphasis was placed on exploring the effect of environmental manipulation inside greenhouses on the *ET*_*C*_ coping with severe water scarcity in the semiarid Mediterranean region The concept of a “decoupling factor”, the omega factor “Ω”, which was analyzing the interactions between the atmosphere and the crop was first described for open field crops, (Jarvis and McNaughton, 1986). Within greenhouses “Ω” is applied as a tool to evaluate the evaporation processes concerning the heat and mass balance as affected by greenhouse climatic equipment. Transpiration was found to be directly affected by the rate of greenhouse ventilation as it plays a key role in establishing the inside microclimate, rather than the radiative components (Baille, 1999; Baille et al., 2005). Hourly variation of Ω was also evaluated by others as a means of increasing the water use efficiency in relation with the appropriate greenhouse climatic equipment (Katsoulas et al.,^ 2015^; Tang and Li, 2017). 

Today, there is no universal method to determine *ET*_*C*_ in a greenhouse even though several models have been used under Mediterranean climatic conditions after recalibration and validation (Karaca et al. 2018; Katsoulas and Stanghellini 2019). However, for an efficient water-saving strategy, the response of *ET*_*C*_ to varying environmental conditions must be known on a minute time scale (Meijer et al. 1985). Considering that the greenhouse soil is many times covered by a plastic sheet, thereby eliminating evaporation from the soil surface, the terms evapotranspiration and transpiration are often used interchangeably (Bartzanas et al. 2005; Takakura et al. 2009).

*ET*_*C*_ could be calculated by the two-step climate method approached (FAO56 methodology) by multiplying reference evapotranspiration (*ET*_*O*_; i.e., the evapotranspiration rate from a reference surface, not short of water) by crop coefficient (*K*_*C*_) values (Allen et al. 1998). In particular, *ET*_*O*_ reflects the atmospheric demand for water from the crop-soil system and *K*_*C*_ characterizes how the crop is modulated that demand (Incrocci et al. 2020). In the past years, a lot of research has been conducted in the field, for computing standard *K*_*C*_ corresponding to a fixed length crop stage (i.e., ground coverage percentage) based on the ratio of *ET*_*C*_ to *ET*_*O*_ during various growth stages. Those values enable the transfer of standard *K*_*C*_ values among locations and climates (Allen et al. 1998; Rallo et al. 2021) especially for perennials crops, under the assumption that *ET*_*O*_ accounts for nearly all variation caused by weather and climate (Allen and Pereira 2009). However, in herbaceous greenhouse crops, it is not easy to obtain similar *K*_*C*_ patterns considering the variability in planning days, growth rate, pruning and harvesting phases, and greenhouse microclimatic conditions. Therefore, the standard FAO method of calculating *ET*_*C*_ using constant *K*_*C*_ values corresponding to a fixed-length crop stage is not recommended in all cases (Allen et al. 1998). Blanco and Folegatti (2003) have shown that the cucumber crop yield is significantly affected by using a fixed value in any specific phenological stage; thus, resulting in an over or underestimation of the water volume to be applied. To overcome this problem, some researchers estimated *K*_*C*_ values as a fraction of thermal time inside the greenhouse daily (Orgaz et al. 2005) while others suggested *K*_*C*_ values based on observations of ground cover and vegetation index (Pereira et al. 2021). The primary characteristics, which distinguished each crop from the reference crop, are the height of the crop and albedo as affected by the fraction of ground cover, the canopy resistance, and evaporation from exposed soil (Allen et al. 1998).

In another case, vertically supported crops such as tomtoes, sweet pepper, green beans, and melons indicated higher *K*_*C*_ values compared with the same outdoor crops (Orgaz et al. 2005). In a recent review article, Incrocci et al. (2020) reported cucumber initial value of *K*_*C*_ equal to 0.2 and a maximum *K*_*C*_ value of 1.2 under Mediterranean climatic conditions. In another case, cucumber *K*_*C*_ values ranging from 0.6 (blooming stage) to 0.9 (height of the wire) were reported (Kittas 1990). Notably, growing conditions and irrigation scheduling could affect among others the *K*_*C*_ values (Allen et al. 1998; Blanco and Folegatti 2003). To the best of our knowledge, little work has been done on the estimation of *K*_*C*_ values related to soilless-based systems in which precise irrigation scheduling is fundamental (Tüzel et al. 2006), particularly considering the limited water holding capacity of the substrates. Thus, the need to improve irrigation scheduling in intensive production systems such as soilless culture requires a good insider into the variability of the *K*_*C*_ especially in water-scarce areas like in the Mediterranean basin Greenhouse design and environmental control equipment influence in a unique way the greenhouse microclimate and consequently the air temperature distribution and humidity (Kittas and Bartzanas, 2007). Modifying the greenhouse microclimate through ventilation and air circulation systems affects crop aerodynamic and canopy resistance values and transpiration (Bartzanas et al., 2004). This research work refers for the first time an hourly different greenhouse soilless-base growth cucumber *K*_*C*_ values cultivated under the same latitudes and cropping conditions; however, under different microclimatic conditions as affected by the greenhouse climatic equipment. Indeed, the *K*_*C*_ factor serves as an aggregation of the physical and physiological differences between crops and the reference definition and may vary during the growing period or even hourly based as in the case of greenhouses.

Τhe FAO56 Penman-Monteith (P-M) equation has been generally recommended as the standard method for the definition and computation of *ET*_*O*_ in the calculation procedures of *K*_*C*_ parameters. On the other hand, P-M equation model requires many climatic data to be available; thus, several researches used others’ *ET*_*O*_ models (e.g., FAO Radiation; Hargreaves; Priestley-Taylor) with reduced climatic inputs (Fernández et al. 2010; Zi-kun et al. 2010; Karaca et al. 2018; Yan et al. 2019). This is the case, for medium technology plastic greenhouses, in the Mediterranean region (Fazlil Ilahi 2009; Yan et al. 2021), using simplified ET_O_ equations after local recalibration (Pereira et al. 2021).

One of the most precise among the simplified methods for estimating reference evapotranspiration is the Priestley-Taylor (P-T) model, which uses, as input data, air temperature and solar radiation values (Valdés-Gómez et al. 2009; Aschonitis et al. 2015). In a recent study, the P-T model was recommended as the first choice to compute *ET*_*O*_ reference evapotranspiration in a greenhouse (Gong et al. 2021, 2020b). In addition, to content, the P-T *α* coefficient was considered to take a constant value of 1.26 within greenhouses (Liu et al. 2008; Karaca et al. 2018). However, it has been documented that this empirical coefficient necessitates recalibration according to the surrounding environment. Particularly, Nikolaou et al. (2022) reported cucumber *α* coefficient values ranging between 0.72 to 0.86 under Mediterranean greenhouse conditions.

The objective of this study was the determination of soilless cucumber crop coefficients grown under different greenhouse microclimatic conditions, quantifying evapotranspiration, and reference evapotranspiration values. The findings may be an efficient tool for irrigation scheduling and better water management in Mediterranean greenhouse horticulture.

## Materials and methods

### Experimental setup

**Fig. 1:**
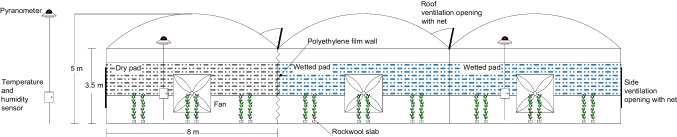
Cross-sectional view of the experimental greenhouse.

Experiments were carried out in a climate-controlled plastic greenhouse at the Agricultural Research Institute, in the coastal area of southern Cyprus (34° 94' N, 33° 19' Ε, altitude 40 m). the coastal area of southern Cyprus. The greenhouse had a total covered area of 504 m^2^ (24 m in length and 21 m in width), and the roof slopes were north-south oriented. The ridge height was 5.0 m, and the gutters' height was 3.50 m. The greenhouse cover was a low-density anti-drip clear polyethylene film with 88% thermal efficiency and global light transmission and 55% diffused light transmission. The greenhouse soil was covered with a double side plastic film (black downwards and white upwards). The greenhouse was equipped with a single continuous roof vent in the middle span for natural ventilation with a maximum opening area of 24 m^2^ (24 m in length and 1 m in width). The side vent was 18 m long and 2.2 m wide with a maximum opening area equal to 52.8 m^2^ at one wall. The roof inlet openings and the sidewall were covered with an insect-proof screen. Dynamic ventilation was performed by three fans, one at each span. The air flow rate of each fan was 31,500 m^3^ h^−1^. The evaporative wet pad cooling system was a 0.15-m-thick cellulose pad located on the south gable end wall that was 14 m in width and 1.2 m in height, with the bottom edge 1.3 m above ground level. Fans and cooling started operating when the greenhouse air temperature exceeded 27 °C. The greenhouse was divided into two compartments by a polyethylene film wall. One compartment was evaporative cooled (active fan-and-pad system; cooled greenhouse compartment), whereas in the other only the exhaust fans were operated (forced ventilation system; uncooled greenhouse compartment) (Fig. 1).

Two subsequent experiments were conducted for computing cucumber crop coefficient *K*_*C*_ in two cropping seasons. During the spring-summer season (April to June) white paint was applied in the uncooled greenhouse, for reducing the incoming solar radiation, following the common practice in local commercial greenhouses. The plastic cover in the cooled greenhouse remained clear. In the following autumn-winter season (October to December) whitewash was removed. The next year during the spring-summer season (April to June) a validation experiment was performed.

Cucumber seeds (*Cucumis sativus* L.) cv. ‘Phenomenon’ were sown on rockwool starter blocks (10 × 10 × 6.5 cm) and transplanted at the stage of two–three true leaves into 15 L (100 cm × 20 cm × 7.5 cm) rockwool slabs (Grodan Company, Denmark), resulting in a plant density of 1.6 pl. m^−2^ (*n* = 360) in all experiments. The plants were prepared two to three weeks before the above said starting experimental period Polyethylene twine was attached 2.2 m above the plant row on a horizontal wire for supporting the plants. Plants were trained to a vertical cordon and pruned according to the umbrella system. Twelve days after transplanting, plants were irrigated at a rate of 0.24 L m^−2^ at fixed time intervals using standard grower practices. Then, to maintain the drainage fraction close to 35–40%, the amount of irrigation was increased to 0.32 L m^−2^. The frequency of irrigation was based on solar radiation values following Katsoulas et al. (2006). An irrigation event was triggered by the integral of solar radiation intensity based on a pyranometer (Wm^−2^; sensor pyranometer type TIR-4P; Bio Instruments Company, Chisinau, Moldova) located outside the greenhouse and regularly adjusted as plants grew. Plants were also irrigated at night-time to account for evaporation from the substrate during the dark hours, thus preventing the substrate from drying out (Beeson 2011). Complete nutrient solution following the stage of development was supplied to the crop according to Savvas and Neocleous (2019).

### Measurements

A set of climatic data, net solar radiation (*R*, W m^−2^), relative humidity (RH, %), and air temperature (Ta, °C) outside and inside each greenhouse were recorded from a meteorological station at 30-s interval (Galileo controller; Galcon, Kfar Blum Israel), and a 10-min average was calculated. Vapor-pressure deficit (VPD) was estimated based on greenhouse air temperature and relative humidity (Allen et al. 1998). 

**Fig. 2:**
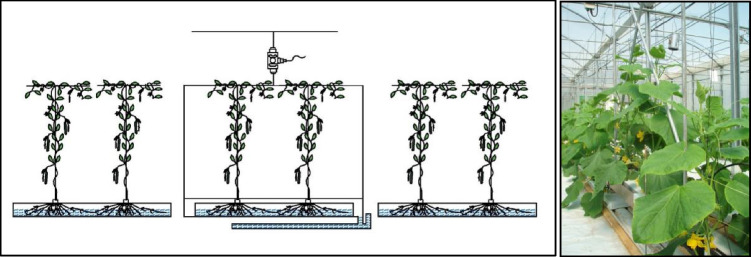
A schematic drawling (right hand side) and a photo (left hand side) of the lysimeter

The transpiration of the crop was measured using weighting-drainage lysimeters (Fig. 2). Rockwool slabs with two plants in each climatic treatment were placed on a platform, consisting of a plant supporting system onto which the plant was trained rather than the crop wires and a drainage water collector system. The whole platform was mounted on the greenhouse ceiling from a suspension load cell allowing the media bag to be at the same height as the other surrounding bags. These scales (Model 9363; Vishay Precision Group, Malvern, PA, USA) have a maximum capacity of 50 kg and a resolution of 0.02 g. The weight loss was measured at 30-s interval by the electronic balance, and a 10-min average was calculated which was assumed to be equal to crop transpiration.

A water balance equation was used as an indirect method to calculate crop water uptake (i.e., crop transpiration) following Eq. (1) (Chen et al. 2013). The drainage water from the lysimeter platform system and in each climatic treatment was automatically collected (a drainage sample machine “Dagan”, compatible with Galileo controller; Galcon, Kfar Blum Israel) by the end of a day and before the first irrigation event and four times daily, at fixed interval times.

1 $$WU=P+I-\textrm{S}-D-R$$

where *WU* is the crop water uptake, *P* is the precipitation, *I* is the irrigation, *S* is the change in soil water storage, *D* is deep percolation, and *R* is runoff (values are expressed in millimeter).The values of *P* and *R* were zero because the experiment was carried out in containers under controlled greenhouse conditions, and *S* is very small because the substrate is continuously close to field/container capacity because of high frequency irrigation (Incrocci et al. 2020).

A series of non-destructive measurements (i.e., plant height, length, and width of each leave) was made in 12 plants (6 labeled and 6 randomly) selected per climatic treatment. The leaf area index (i.e., the green leaf area of cucumber plants per unit area (A); LAI: m^2^ leaf m^−2^ ground) was derived from a single relationship as a function of length (L) × with (W) product (i.e., A=1.629L * W) following Nikolaou et al. (2017). The measures were performed 8 times for growth stages I to IV during the experimental period.

- Stage I: Initial stage from planting date to approximately 10% of ground cover.
- Stage II: Development stage which corresponds to the initiation of flowering and effective full cover.
- Stage III: Mid-season stage which runs from full cover to the start of maturity as indicated by leaves yellowing or senescence of leaves.
- Stage IV: Late-season stage runs from maturity to full senescence.

### Priestley and Taylor model parameterization

A Priestley-Taylor model (Eq. (2)) was used to compute *λE*_*O*_ on a 10-min time step basis in each climatic treatment using the net radiation values, the slope of the saturated vapor pressure-temperature, and assuming that soil heat flux advection is considered negligible (Liu et al. 2008; Karaca et al. 2018; Gong et al. 2020b; Ghiat et al. 2021).

2 $$\lambda {ET}_O=\alpha \frac{\Delta}{\Delta +\gamma}\left({R}_n-G\right)$$

where *λ* is the heat of water vaporization (J kg^−1^); *λET*_*O*_ is the latent heat flux (W m^−2^); *α* is the P-T coefficient (dimensionless); Δ is the slope of the saturated vapor pressure-temperature curve (kPa °C^−1^), *γ* is the psychometric constant (kPa °C^−1^); Rn is the net radiation (W m^−2^ day^−1^); *G* is the soil heat flux (W m^−2^ day^−1^; relatively small and usually neglected). The P-T *α* coefficient was estimated, based on greenhouse mean air temperature (*T*_mean_; °C) values following Nikolaou et al. 2022 (Eq. (3)):

3 $$\alpha =\frac{\left(\varDelta +\gamma \right)}{\varDelta}\lambda\ 0.0135\ \left({T}_{\textrm{mean}}+17.8\right)$$

### Crop coefficient estimation, and ET_C_ model validation

The methodology proposed by FAO (Allen et al. 1998) to calculate crop evapotranspiration considers separately the effects of the climate (reference evapotranspiration-*ET*_*O*_) and the plant canopy (crop coefficient, *K*_*C*_) on the crop’s water consumption. The crop’s evapotranspiration is thus calculated by the following equation:

4 $${ET}_C={K}_C\times {ET}_O$$

where *K*_*C*_ is the crop coefficient (dimensionless). Therefore, the crop coefficient *K*_*C*_ can be computed from the ratio between crop evapotranspiration measurements and reference evapotranspiration calculations. Evapotranspiration could express in terms of water loss in millimeter or in terms of energy received per unit area required to vaporize free water (i.e., latent heat of vaporization *λΕΤ*) which is a function of the water temperature (at 20 °C, *λ* is about 2.45 MJ kg^−1^; 1 mm is equivalent to 2.45 MJ m^−2^ (Allen et al. 1998). The *K*_*C*_ values were calculated on a 10-min time interval and averaged for each cucumber growing stage corresponding to stages I to IV.

Regression analysis between calculated crop coefficients and growth parameters (i.e., plant leaf area index) was performed as an attempt of replacing *K*_*C*_ in Eq. (4) by easily obtained crop measurements. To test the representations of the relationship developed to predict the *K*_*C*_ values, simulated *λΕΤ*_*CS*_ based on *λΕΤ*_*Ο*_ estimated by the Priestley and Taylor model, validated against *λΕΤ*_*CM*_ (as measured with the lysimeters) in another experiment.

### Statistical analysis

Data were analyzed and comparisons of means were tested with ANOVA using a Statistical Package for the Social Sciences (IBM Corp. Release 2011. IBM SPSS Statistics for Windows, Version 20.0. Armonk, NY, USA: IBM Corp). Regression analysis was performed for the estimation of relationships between selected data.

## Results

### Climatic conditions and crop coefficient

In the spring-summer season, the mean external daily climatic conditions, in the spring-summer season, were for solar radiation 694 W m^−2^, air temperature 30 °C, and relative humidity 52 %. During the autumn-winter season, the solar radiation was 436 W m^−2^, air temperature 24 °C, and air humidity 58%. As expected, a higher internal humidity was observed in both growing seasons within greenhouses.

As in Table 1, the mean indoor daily climatic conditions (temperature, relative humidity, and radiation) are given separately according to the growth stages of the crop. Inside the greenhouse climatic parameters were found to have significant differences (*p* ≤ 0.05) between the uncooled and cooled compartments in both growing periods. Higher air temperature and VPD values were recorded in the uncooled compared with the cooled greenhouse compartment, irrespective of the growth period (Table 1). Particularly, increased air temperature up to 3 °C was recorded in the uncooled greenhouse during the spring-summer season, despite that whitewash reduced the incoming solar radiation by 109 W m^−2^.

**Table 1:** Mean daily values (±standard error) of inside climatic data, plant growth, and crop coefficient values for daylight hours during cucumber crop development stages in the spring-summer and autumn–winter growing period

Treatment	Stages	*RGi*	*VPDi*	*Ti*	*RHi*	*λEΤ*_*C*_	*λETo*	*α*	*K*_*C*_
Spring-summer season									
Uncooled	I	377 (16.9)	1.8 (0.06)	29 (0.2)	57 (12.3)	154 (15.5)	281 (12.9)	0.817	0.55 (0.05)
	II	402 (7.8)	1.9 (0.03)	30 (0.1)	59 (10.8)	162 (5.0)	276 (5.8)	0.818	0.59 (0.02)
	III	425 (9.15)	1.8 (0.05)	37 (0.2)	63 (12.7)	189 (8.2)	278 (9.6)	0.837	0.68 (0.01)
	IV	427 (18.2)	2.8 (1.8)	32 (0.2)	60 (7.4)	172 (3.5)	265 (4.2)	0.826	0.65 (0.03)
	AVG	408 (5.3)	2.1 (0.02)	32 (0.1)	60 (8.8)	176 (2.7)	271 (3.1)	0.825	0.65 (0.01)
Cooled	I	478 (21.4)	1.5 (0.02)	28 (0.1)	61 (9.1)	115 (14.4)	276 (12.4)	0.805	0.42 (0.04)
	II	509 (9.9)	1.6 (0.07)	27 (0.11)	64 (8.9)	138 (4.2)	271 (5.6)	0.806	0.51 (0.01)
	III	540 (11.6)	1.5 (0.2)	34 (0.1)	67 (9.8)	225 (9.1)	275 (9.5)	0.826	0.82 (0.01)
	IV	542 (23.1)	1.6 (0.1)	30 (0.3)	60 (6.1)	174 (3.6)	260 (4.1)	0.812	0.67 (0.03)
	AVG	517 (6.7)	1.6 (0.01)	29 (0.08)	63 (8.1)	170 (2.8)	266 (3.1)	0.812	0.64 (0.02)
Autumn-winter season									
Uncooled	I	346 (7.4)	2.1 (0.03)	30 (0.2)	49 (3.8)	107 (15.4)	171 (14.5)	0.827	0.63 (0.1)
	II	274 (7.8)	1.7 (0.03)	29 (0.02)	56 (11.1)	115 (6.4)	167 (6.4)	0.806	0.69 (0.03)
	III	235(15.0)	1.2 (0.07)	26 (0.6)	69 (12.5)	159 (8.1)	173 (6.8)	0.804	0.92 (0.04)
	IV	185 (29.2)	0.6 (0.08)	21 (0.5)	68 (8.6)	125 (10.9)	161 (6.9)	0.796	0.78 (0.04)
	AVG	260 (4.9)	1.4 (0.02)	27 (0.01)	60 (8.9)	128 (4.7)	167 (3.7)	0.804	0.77 (0.02)
Cooled	I	346 (7.4)	1.6 (0.02)	27 (2.8)	57 (13.3)	98 (13.5)	161 (13.4)	0.804	0.61 (0.4)
	II	274 (7.8)	1.1 (0.18)	24 (0.1)	61 (8.1)	111 (6.5)	151 (5.7)	0.772	0.74 (0.6)
	III	235(15.0)	0.8 (0.05)	21 (0.4)	65 (15.2)	143 (6.9)	154 (5.7)	0.763	0.93 (0.6)
	IV	185 (29.2)	0.7 (0.08)	21 (0.6)	67(9.4)	105 (9.5)	142 (5.6)	0.758	0.74 (0.8)
	AVG	260 (4.9)	1.1 (0.01)	23 (0.1)	62 (11.0)	116 (4.2)	149 (3.2)	0.768	0.78 (0.2)

Stages (crop development stage I, initial, II development, III, middle, IV, late, AVG, Average); RGi, inside greenhouse solar radiation (W m^−2^); VPDi, inside greenhouse air vapor pressure deficit (kPa); Ti, inside greenhouse air temperature (°C); RHi, inside greenhouse air relative humidity (%); λEΤ_C,_ mean daily crop evapotranspiration (W m^−2^); λEΤ_O_, mean daily reference evapotranspiration (W m^−2^); *α*, the P-T *α* coefficient; Kc, crop coefficient (dimensionless)

The results presented in Table 1 showed that the mean *λETo* value was higher in the uncooled greenhouse compartment. Especially autumn-winter period showed differences of *λETo* between compartments ranging up to 12%. The *λETo* ranged between 260 and 281 W m^−2^ during the spring-summer period and between 142 and 173 W m^−2^ during autumn-winter. In absolute values, the P-T *α* coefficients were higher during the spring-summer period in the uncooled (0.83) than during autumn-winter and in the cooled greenhouse compartment (0.81).

The mean daily values of *λET*_*C*_ followed quite a similar trend for different crop development stages in the two compartments (cooled and uncooled), in both growing periods as may be expected. Slightly higher *λET*_*C*_ was recorded in the uncooled greenhouse part during the autumn-winter period. Thus, the mean daily water uptake calculated in a week representative period (stage III) was estimated at 3.9 L m^−2^ (uncooled) and 2.5 L m^−2^ (cooled) m^−2^ (cooled) in autumn-winter. However, during the spring-summer season, higher *ET*_*C*_ values were not always recorded in the uncooled treatment. Especially in stage III, as the crop growth, estimated *ET*_*C*_ values were by 19% (36 W m^−2^) higher for the cooled treatment.

**Fig. 3:**
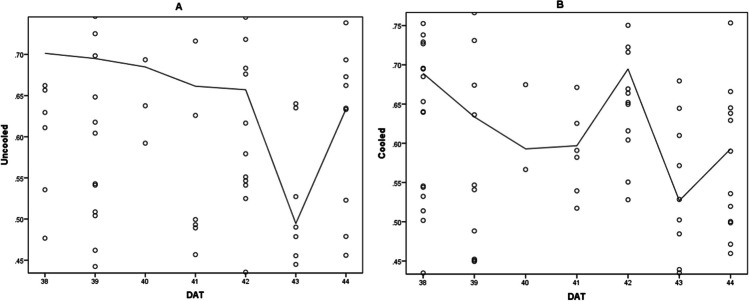
Cucumber crop coefficient values from DAT 38-45 in **A** uncooled and **B** cooled greenhouse compartment during the spring-summer growing period

**Fig. 4:**
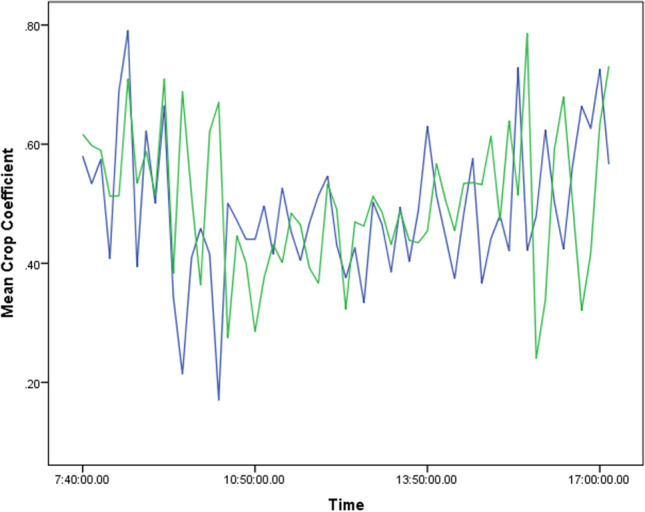
Hourly variation of cucumber crop coefficient during the autumn-winter growing period in an uncooled greenhouse-blue line and in a cooled greenhouse-green line

The results presented in Table 1 showed that average growth-stage-specific *K*_*C*_ values have different values under different greenhouse microclimatic conditions. However, during the autumn-winter period, higher *K*_*C*_ values were obtained (avg, 0.78) compared to the spring-summer (avg, 0.65) due to the degree of defoliation. Thus, the highest *K*_*C*_ value observed was 0.93 in the autumn-winter and the lowest was 0.51 in the spring-summer cycle. However, Figs. 3 and 4 show that *K*_*C*_ values vary considerably also on a weekly, daily and sub-daily basis. On DAT (Day After Transplant) 43 the *K*_*C*_ decline observed for both climatic treatments can be explained by intense defoliation.

The mean hourly *K*_*C*_ variations in the greenhouse compartment during the autumn-winter period are illustrated in Fig. 3. It can be observed that there is a tendency for *K*_*C*_ values to decrease during midday hours.

Higher values of plant height and leaf area index LAI were found when the greenhouse remained uncooled irrespectively of the growing period (Table 2). This corresponds to an increase, for plant height over a season, by 16 for plant height and by 8-11 for LAI. Notably, higher values of LAI and plant height were estimated in all stages of growth in the uncooled than in the cooled greenhouse compartment.

 However, higher values of plant height were not always associated with higher values of LAI (Table 2).

**Table 2:** Mean values of crop growth parameters

		Spring-summer season		Autumn-winter season	
Treatment	Stages	H	LAI	H	LAI
Uncooled	I	76.7 (1.1)	0.73 (0.03)	53.9 (4.3)	0.44 (0.05)
	II	153.6 (1.9)	1.35 (0.07)	175.2 (11.7)	1.45 (0.2)
	III	222.6 (7.8)	1.63 (0.07)	248.0 (15.3)	1.78 (0.1)
	IV	229.0 (3.5)	1.79 (0.04)	270.9 (22.7)	1.51 (0.1)
	AVG	170.4 (10.5)	1.37 (0.08)	187.0 (17.3)	1.29 (0.2)
Cooled	I	70.2 (1.4)	0.63 (0.02)	45.8 (5.1)	0.35 (0.07)
	II	135.0 (3.5)	1.40 (0.1)	145.6 (8.9)	1.16 (0.2)
	III	182.0 (3.7)	1.28 (0.1)	208.9 (12.3)	1.63 (0.2)
	IV	188.1 (4.4)	1.77 (0.04)	229.4 (9.8)	1.49 (0.2)
	AVG	143.8 (8.2)	1.26 (0.09)	157.4 (7.5)	1.15 (0.3)

Stages (crop development stage I, initial, II development, III, middle, IV, late, AVG, Average); H, plant height (cm); LAI, leaf area index (m^2^ leaf m^-2^ ground)

### Model parameterization and validation

To determine the relationship between crop coefficient *K*_*C*_ and growth parameters (i.e., plant leaf area index), non-linear regression analysis was performed on data derived from both greenhouse compartments. *K*_*C*_ and leaf area index showed very high correlation coefficients for linear, quadratic, and cubic regressions. The coefficients of determinations *r*^2^ for both quadratic and cubic equations were 0.91 and 0.96, respectively. However, the linear relationship between variables studied (i.e., *K*_*C*_ = 0.447 × LAI) showed also a very high coefficient *r*^2^ = 0.91 which allow us to proceed with the validation procedure maintaining simplicity as previously suggested (Williams and Ayars 2005; Pereira et al. 2021). Therefore, the cucumber evapotranspiration could be estimated considering Eqs. (2) and (4), as below:

5 $$\uplambda {\textrm{ET}}_{\textrm{C}}=0.447\ LAI\ \alpha \frac{\Delta}{\Delta +\upgamma}\ Rn$$

For validation purposes, the cucumber transpiration was simulated (i.e., *λEΤ*_*CS*_) the following year. A linear regression analysis between *λEΤ*_*CS*_ (simulated) and *λEΤ*_*CM*_ (measured) was applied for estimating the coefficient of determinations (*r*^2^), the intercepts (*a*), and slopes (*b*) for specified time intervals (DAT 15, 30, 45, 60). The *r*^2^ values were statistically significant (*P* < 0.05) ranging from 0.46 to 0.86 for the different growth stages (Table 3).

**Table 3:** The linear regression coefficient of determination (*r*^2^), between evapotranspiration, simulated (*λEΤ*_*S*_) and measured (*λEΤ*_*M*_) by lysimeter under greenhouse conditions in a spring-summer growing period

DAT	LAI	*r*^2^	*B*	Se	Sig.
15	0.09	0.46	0,67	1.97	0.004
30	0.5	0.77	0.87	0.25	0.000
45	0.98	0.83	0.914	0.14	0.000
60	0.68	0.84	0.915	0.11	0.000

*r*^*2*^ correlation coefficient, *b* beta value/slope, *se* standard error

During validation, the mean indoor climatic conditions were greenhouse air temperature 23.8 (3.2) °C, VPD 1.4 (0.4) kPa, and relative humidity 49.9 (6.4) % correspondingly. The mean indoor solar radiation was 442 W m^−2^.

## Discussion

Precise irrigation scheduling based on environmental greenhouse data involves consideration of crops’ coefficient values. Hourly crop coefficients are needed to optimize the water application efficiency of high-frequency irrigation systems, even though they are rarely reported in the literature (Irmak et al. 2013). Research over the past years has demonstrated that growth stage-specific *K*_*C*_ values are also site-specific as they are sensitive to changes in air vapor density, air temperature, wind speed, and to a lesser degree to solar radiation (Annandale and Stockle 1994; Čerekovic et al. 2010; Mulovhedzi et al. 2020; Ávila-Dávila et al. 2021). Particularly, the primary limitation in semi-arid regions for estimating greenhouse *K*_*C*_ values is related to the energy and mass fluxes of the greenhouse atmosphere. That is because are constantly varied and affected by the outside conditions and greenhouse environmental control systems. This effect is more pronounced in regions with yearly round high heat loads where the greenhouses could not keep closed for long periods, as in the case of northern European greenhouses. Manipulating the greenhouse environment through the proper management of the climate control equipment such as cooling, ventilation, and shading entails a direct effect on crop evapotranspiration thereby, crop water consumption.

In this study, we defined variations of cucumber crop coefficient values under different greenhouse microclimatic conditions. The results obtained show that growth-stage-specific *K*_*C*_ values vary considerably on a weekly, daily, and sub-daily basis between climatic treatments tested. The present results clearly show that in a Mediterranean greenhouse, the evolution of cucumber *K*_*C*_ values in soilless culture followed a similar pattern to that of field-grown vegetables crops however, different values were defined within stages of growth further adjusted to different greenhouse microclimates (Table 1). After the initial growth stage I, *K*_*C*_ rabidly increased and reached maximum values in stage III which define the mid-season stage. Finally, a slight decline of *K*_*C*_ observed at the end of crop cycles (stage IV; late-season stage) normally attributed to leaf senescence.

In our case, the seasonal *K*_*C*_ during spring-summer period varied from 0.51 to 0.82 and during autumn-winter from 0.61 to 0.93. The averaged *K*_*C*_ values obtained under our experimental conditions were lower than those estimated for a soilless cucumber crop in a humid tropical climate (*K*_*C*_ = 1.25; Salcedo et al. 2017), and in any case, *K*_*C*_ did not reach maximum values obtained in another Mediterranean region (0.93 vs. 1.2; Incrocci et al. 2020 a). However, they were very close to those obtained for cucumber crops grown in an area facing also an intense Mediterranean climate (Kittas 1990). The different cucumber crop coefficient values found in the literature are summarized below (Table 4):

**Table 4:** Cucumber crop coefficient values in different growing conditions

Cropping conditions	Cucumber crop coefficient	References
Soilless-based greenhouse crop; autumn-winter growing period, 2 plants m^−2^	*K*_*C*_ initial = 0.2 and *K*_*C*_ max. = 1.2	Fernandez et al. (2001)
Soil-based greenhouse crop in a Mediterranean area	*K*_*C*_ from planting to blooming = 0.6; *K*_*C*_ from blooming to the height of the wire from 0.6 to 0.9; *K*_*C*_ harvest = 0.9	Kittas et al. (1990)
Soilless-based greenhouse crop in a humid-tropical area; autumn-winter growing period, 1.3 plants m^−2^	Average *K*_*C*_ = 1.25	Salcedo et al. (2017)
Soil-based greenhouse crop; winter-spring growing period in Brazil, 2.5 plants m^−2^, different irrigation salinities tested	*K*_*C*_ mean maximum in different treatments = 1.26 to 1.49 *K*_*C*_ late season stage in different treatments = 0.44 to 0.73	Blanco and Folegatti (2003)
Soil-based greenhouse crop (cv Maram) in a Mediterranean area	*K*_*C*_ end = 1.2	Eliades (1988)
Open-field crop in a sub-humid climate	*K*_*C*_ initial = 0.5; *K*_*C*_ mid-development = 1; *K*_*C*_ end = 0.8	Allen et al. (1998)
Soil-based greenhouse crop in arid conditions, Iran	*K*_*C*_ initial = 0.19; *K*_*C*_ development = 0.64; *K*_*C*_ mid-season = 0.99; *K*_*C*_ end = 0.81	Rezaverdinejad et al. (2017)
Soil-based greenhouse crop in arid conditions, Iraq	*K*_*C*_ initial = 0.16; *K*_*C*_ development = 0.87 to 1.23; *K*_*C*_ end = 0.87	Hamaza and Almasaf (2015)
Soil-based greenhouse crop in arid conditions, Iraq	*K*_*C*_ initial = 0.1; *K*_*C*_ mid-development = 1.29; *K*_*C*_ end = 0.87	Sadoon (2020)

The intensity of defoliation, which is of common cultivation practice for cucumber, has a direct effect on LAI and thereby crop coefficient values. Although maintaining a higher value of LAI will help to alleviate the exceed heat stress in semi-arid conditions, the indoor climate conditions are many times favorable for cucumber fungus infestations. Thus, under such conditions defoliation is mandatory to reduce pesticide applications and maintain healthier plants (Baudoin et al. 2013). It is acknowledged that in the present paper, we do not mention yield data with regard to climatic treatments. The effect of climate on cucumber crop growth and yield cucumber under different greenhouse microclimatic conditions was examined by Nikolaou et al. (2018, 2019). Briefly, the authors’ showed that the absence of an active cooling system, cucumber crop can grow and produce without yield restrictions even throughout the spring-summer crop period of the year. In addition, the forced air ventilation system proved to be capable of preventing the overheating of a crop due to the fact that high transpiration rates (due to higher LAI) decrease the ambient–inside greenhouse air differences up to 3.2 °C.

Since water scarcity is one of the main problems for greenhouses located in semi-arid regions of the Mediterranean, precise irrigation scheduling will improve water application efficiency. For example, we can conclude that an extra amount of 1.25 mm of irrigation is needed daily if we use *K*_*C*_ recommended values for the later stages of growth (autumn-winter period, Stage III, 30 days) in soilless cucumber crops (*K*_*C*_ value of 1.2; Table 4 ; Fernandez et al. 2001) compared to the suggested *K*_*C*_ value (*K*_C _= 0.93). The higher average transpiration values observed in the uncooled treatment in both growing periods, compared well with previous observations related to the greenhouse climate and crop development (Tables 1 and 2) (Baille 1999). There were considerable differences in the transpiration values within crop stages and between climatic treatments. Particularly, as cucumber plants increase in size, the increase in *λET*_*C*_ values observed was directly related to higher *K*_*C*_ values. In another case, Irmak et al. (2013) stated that while the *K*_*C*_ approach can predict daily transpiration values with varying degrees of accuracy for low-frequency irrigation applications, scheduling irrigation events at a higher frequency, such as the soilless-based cultures, is best to accomplish using hourly *K*_*C*_ values. Indeed, data in Fig. 4 coincide with these findings. Additionally, according to Baille et al. (2005), low values of “Ω”, detected in Mediterranean greenhouses under summer conditions, underline the primacy of VPD and stomatal canopy conductance in determining the level of canopy transpiration rate. Therefore, it appears that the hypothesis that transpiration of greenhouse crops is mainly driven by the radiative component, suggested by several authors and validated for closed or poorly ventilated greenhouses, should be revised when dealing with summer climate control or greenhouse design in warm and dry climates.

Another factor correlated to *λET*_*O*_ values is the variation in wind speed which alters the aerodynamic resistances, especially for crops that are taller than the hypothetical grass reference (Allen et al. 1998). In our case, an excessive air exchange rate was observed in the uncooled greenhouse as affected by the continuous operating of the exhaust fans. It is well known that increasing wind speed make latent heat exchange more efficient and reduced the crop canopy temperature (Annandale and Stockle. 1994). In fact, wind speed in the two treatments is not very different when the fans work. When the fans are not working air speed in the greenhouse is reduced. Therefore, given that exhaust fans operate continuously in the uncooled greenhouse and periodically in the cooled greenhouse, the air speed regime above the canopy is greater in the uncooled greenhouse over the day. It is well known that increasing wind speed makes latent heat exchange more efficient and reduced the crop canopy temperature (Annandale and Stockle 1994). In fact, wind speed in the two treatments is not very different when the fans work. When the fans are not working air speed in the greenhouse is reduced. Therefore, given that exhaust fans operate continuously in the uncooled greenhouse and periodically in the cooled greenhouse, the air speed regime above the canopy is greater in the uncooled greenhouse over the day. It is well known that increasing wind speed makes latent heat exchange more efficient and reduced the crop canopy temperature (Annandale and Stockle 1994). This was due to the VPD values which were in all greenhouses in the range of 2 kPa, indicating non-stressed crop conditions (Nikolaou et al. 2020). The critical value of about 2 kPa for air VPD was first mentioned by Baille et al. (1994) as a reference value (or set-point) for actuating the evaporative cooling system (mist system) to avoid stomatal closure. According to this work mean values close to this indicative value of 2 kPa show no stressed conditions for uncooled and cooled greenhouses in Greece. The highest value of VPD reached near 13:00–14:00h and decreased afterward.

The higher *λET*_*C*_ values recorded in the cooled treatment in stage III, with full- canopy- cover, are due to the significantly higher values of stomatal conductance reached under a greenhouse with evaporative cooling systems (Katsoulas et al.,^ 2001^; Baiile et al., 2005).This coincides with the work of (Nikolaou et al. 2020) who reported different aerodynamic and canopy resistance values for cucumber in a greenhouse that was cooled by a forced air ventilation system. 

The findings of this study suggest that the Priestley-Taylor model can be used to estimate *λEΤ*_*O*_ in Mediterranean greenhouses, especially in cases of fewer available climatic inputs. Note, however, that the climatic equipment and common agricultural practices such as whitening affect the rate of *λEΤ*_*O*_. In the autumn-winter crop, the higher average air temperature and VPD observed in the uncooled greenhouse part resulted in an increased level of *λET*_*O*_ compared to the cooled part, which clearly shows the effect of different microclimatic conditions on the rate of *λEΤ*_*O*_. Furthermore, in the autumn-winter growing period despite the higher values of VPD and air temperature in the uncooled greenhouse *λET*_*O*_ was decreased up to 12% in comparison with the cooled greenhouse part, However, average *λET*_*O*_ values were almost the same in the spring-summer (only 1.8% higher in the uncooled compartment), which let us suggest that common agricultural practices such as whitening, affect the rate of *λEΤ*_*O*_. Particularly, there was decrease in solar radiation by almost 22%, which corresponds to an average difference between treatments of 109 W m^−2^. In contrast, Fernández et al. (2010) reported on a 21% reduction in *λET*_*O*_ in a whitened passively ventilated from March to September greenhouse in a Mediterranean region. A decreased of mean solar radiation by 49% from spring-summer to autumn-winter growth cycles (cooled treatment; clear greenhouse cover) resulted in reduced *λEΤ*_*O*_ values by 44%. Mean daily measured *λET*_*O*_ averaged from 268 W m^−2^ (SS) to 158 W m^−2^ (AW). Those values were in agreement which those proposed by other researchers for *E*_*O*_ in arid and semi-arid regions (Allen et al. 1998; Fernández et al. 2010).

This work successfully generated some regression models to estimate cucumber *K*_*C*_ values under Mediterranean greenhouse conditions. Although there is not much information in the literature for using LAI as a base scale in *K*_*C*_ curves (Irmak et al. 2013), our data support a clear relationship between *K*_*C*_ and LAI. These models are easier to use for the prediction of *K*_*C*_, due to the nature of the variables considered as inputs (i.e., LAI) rather than the direct estimation of *K*_*C*_ by reversing the two-step climate method approach. Crop coefficients were computed for each of the four-cucumber growth stage from LAI observations. From Table 3, we can observe that as the LAI value increased, a higher coefficient of determination (*r*^2^) between the evapotranspiration simulated and the measure obtained.

The foregoing shows that expanding our evaluations for increasing water efficiency in semi-arid regions, the simplified procedure for transpiration estimations from simple growth parameters measurements and reference evapotranspiration appears to be well- founded during the validation experimental processes. The *K*_*C*_ variation obtained indicate a more complex response to greenhouse microclimatic conditions; therefore, native approaches to the site-specific crop coefficient factor estimations should be encouraged.

## Conclusion

Using the Priestley-Taylor evapotranspiration model, reference evapotranspiration (*λE*_*O*_) is computed in different greenhouse environments in a semi-arid region. It was found that the greenhouse climatic equipment used directly influence the *λE*_*O*_ even within the same growing period. The *K*_*C*_ values of cucumber growth stages in different climatic treatments were presented from the ratio between *λET*_*C*_ measurements and *λET*_*O*_ calculations. The regenerated evapotranspiration model based on *λET*_*O*_ measurements and crop growth parameters aids determination in precision agricultural systems. Additional research is needed for the parameterization of the Priestley-Taylor method “*α* coefficient” and hourly-based *K*_*C*_ for a given set of greenhouse microclimatic and crop growing conditions.

The following conclusions can be drawn.

1. Based on the obvious greenhouse microclimatic variation of* K*_*C*_, it is recommended not to apply a* K*_*C*_ as a constant for transpiration estimation even at greenhouses located within the same region. The greenhouse climatic equipment used may directly affect the *K*_*C*_ values with hourly or even shorter times intervals during the day.
2. Evapotranspiration of substrate-based cucumber greenhouse crops can be modeled satisfactory based on the proposed relationship between crop coefficient and leaf area index.
3. In the case of limited climatic data, the P-T equation could be used of estimating* ET*_*O*_. However, the greenhouse climatic equipment should have a direct effect on the α coefficient, therefore, it should not be considered a constant.
